# Infected Dentigerous Cyst With Extraoral Fistula in a Child

**DOI:** 10.7759/cureus.99938

**Published:** 2025-12-23

**Authors:** Tuqa A Abdulsalam, Bachir Farzat, Bosaina Otour, Mohamed Bachir Farzat, Rahma Abdulsalam Abdulhameed

**Affiliations:** 1 General Pediatric, Al Jalila Children Hospital, Dubai, ARE; 2 Pediatric Emergency Medicine, Al Jalila Children's Speciality Hospital, Dubai, ARE; 3 General Pediatrics, Al Jalila Children's Speciality Hospital, Dubai, ARE; 4 Dentistry, University Of Sharjah, Sharjah, ARE

**Keywords:** dentigerous cyst, extraoral fistula, impacted tooth, infected dentigerous cyst, mandibular cyst, maxillofacial surgery, odontogenic infection, odontogenic infectionm skin infection, pediatric odontogenic cyst, prevotella oris

## Abstract

Dentigerous cysts are the most common developmental odontogenic cysts. Still, a secondarily infected dentigerous cyst in children that results in an extraoral fistula is rare and can present as a routine odontogenic abscess. In this paper, we present a case of a healthy 10-year-old girl with progressive pain, swelling, and purulent discharge of the left mandibular area documented for one month after outpatient antibiotic treatment. Clinical examination showed a fluctuant facial swelling with an external fistula, while CT revealed a well-defined unilocular cystic lesion with an unerupted mandibular second premolar. The patient was treated with surgical enucleation of the cyst, extraction of the retained second molar and premolar, and excision of the external fistula. The wound culture grew *Prevotella oris*, which informed appropriate anaerobic antibiotic therapy. The cyst was histopathologically proven to be an inflamed dentigerous type. The patient had an uneventful postoperative course and made a complete clinical recovery. This case illustrates the value of early imaging, culture-specific antibiotics, and surgical treatment in children with prolonged jaw swelling or recurrent dental infections to minimize morbidity and mortality.

## Introduction

Dentigerous cysts are frequently occurring developmental odontogenic cysts and represent about 20-24% of the total number of odontogenic cysts of the jaws. These are caused by the accumulation of fluid between the reduced enamel epithelium and the crown of an unerupted tooth, most commonly occurring with mandibular third molars and maxillary canines [1, 2].

Dentigerous cysts can be found at any age, although diagnosis in children is of significant clinical importance, as these lesions may disturb the eruption process and development of the jaw. Untreated lesions can cause malocclusion, cortical bone expansion, or pathological fracture [3, 4]. The majority of cases are asymptomatic and incidentally found on routine radiographic investigation, but larger lesions can be associated with jaw swelling, facial asymmetry, or displacement of the neighboring teeth [5].

Secondary infection is rare but can happen and may present as odontogenic abscesses with pain and swelling. Rarely, chronic infection may lead to extraoral sinus tract formation, making the underlying diagnosis obtuse [6]. Infected odontogenic cysts are often linked to anaerobic bacteria, particularly species of *Prevotella*, *Porphyromonas*, and *Fusobacterium*, and this highlights the necessity of microbiological identification with organ-based antimicrobial therapy in addition to surgical intervention [7, 8].

CT is crucial in assessing the extent of lesions, the involvement of cortical bone, and their relationship to adjacent teeth to help distinguish between other odontogenic pathologies [9]. In pediatric patients, its management varies from marsupialization to enucleation depending upon the size of the lesion, symptoms, and infection [9].

This article reports an unusual case of an infected dentigerous cyst in association with chronic infection, extraoral fistula, and impaction of both a primary and an unerupted permanent tooth in a child.

## Case presentation

A 10-year-old healthy girl presented to the pediatric emergency department with a one-month history of slowly increasing swelling on her left lower jaw. Her symptoms were after the onset of decay and bilateral lower dental abscesses, assessed by a dentist who treated her with oral antibiotics. The child continued to suffer from a chronic left-sided toothache and periodic jaw pain after completing the therapy. The nodule expanded over the next 8 days and became tender, warm, and erythematous, with a drainage of purulent material. The pain was mild, aching by nature, continuous, and exacerbated by chewing and pressure. She was afebrile at this time and had no systemic symptoms, including fever, chills, dysphagia, respiratory distress, drooling, vomiting, poor oral intake, or neck stiffness. There was no trauma, recent upper respiratory infection, sick contacts, or travel.

**Figure 1:**
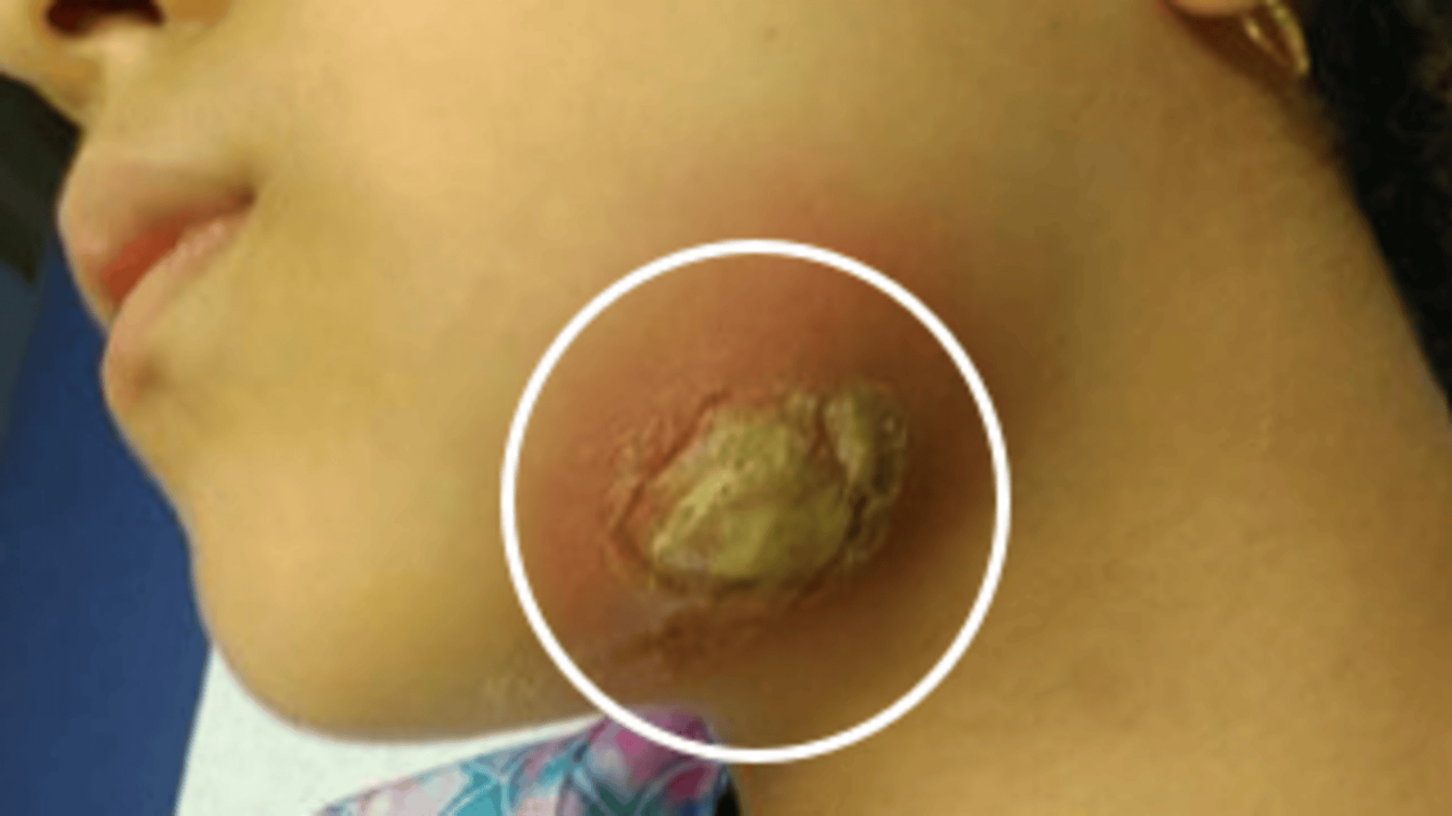
Clinical photograph showing left mandibular swelling with an active extraoral draining fistula. The image demonstrates a well-defined erythematous swelling over the left lower face with a central crusted fistulous opening, consistent with a chronic odontogenic infection draining externally.

At presentation to the emergency department, she was non-toxic and hemodynamically stable. The patient had soft, fluctuant, erythematous swellings of the left mandibular body, with tenderness and purulent discharge from an extraoral fistula (Figure 1).

Intraorally, the patient has a carious tooth with no soft-tissue swelling. Her oral mucous membranes were moist, and she did not have trismus, drooling, airway distress, or cervical adenopathy. The complete blood count (CBC) showed lymphocytosis and eosinophilia. Her inflammatory markers were mildly elevated: CRP (4.3 mg/L) and procalcitonin (0.06 ng/mL). The Gram-stained wound specimen revealed sparse WBCs, scarce epithelial cells, and a small number of Gram-negative rods with 1+ Gram-negative coccobacilli (Table 1). 

**Table 1:** Laboratory Investigations on Presentation

Laboratory Parameter	Result	Reference Range
White blood cell count	10.8 × 10³/μL	4.5–13.5 × 10³/μL
Neutrophils	42%	40–70%
Lymphocytes	46%	20–45%
Eosinophils	8%	0–6%
C-reactive protein (CRP)	4.3 mg/L	< 1 mg/L
Procalcitonin	0.06 ng/mL	<0.1 ng/mL
Serum creatinine	0.4 mg/dL	0.3–0.7 mg/dL
Blood urea nitrogen (BUN)	10 mg/dL	7–20 mg/dL
Alanine aminotransferase (ALT)	18 U/L	7–35 U/L
Aspartate aminotransferase (AST)	22 U/L	10–40 U/L
Wound Gram stain	Few WBCs; Gram-negative rods and coccobacilli	—
Wound culture	Prevotella oris	—

**Figure 2:**
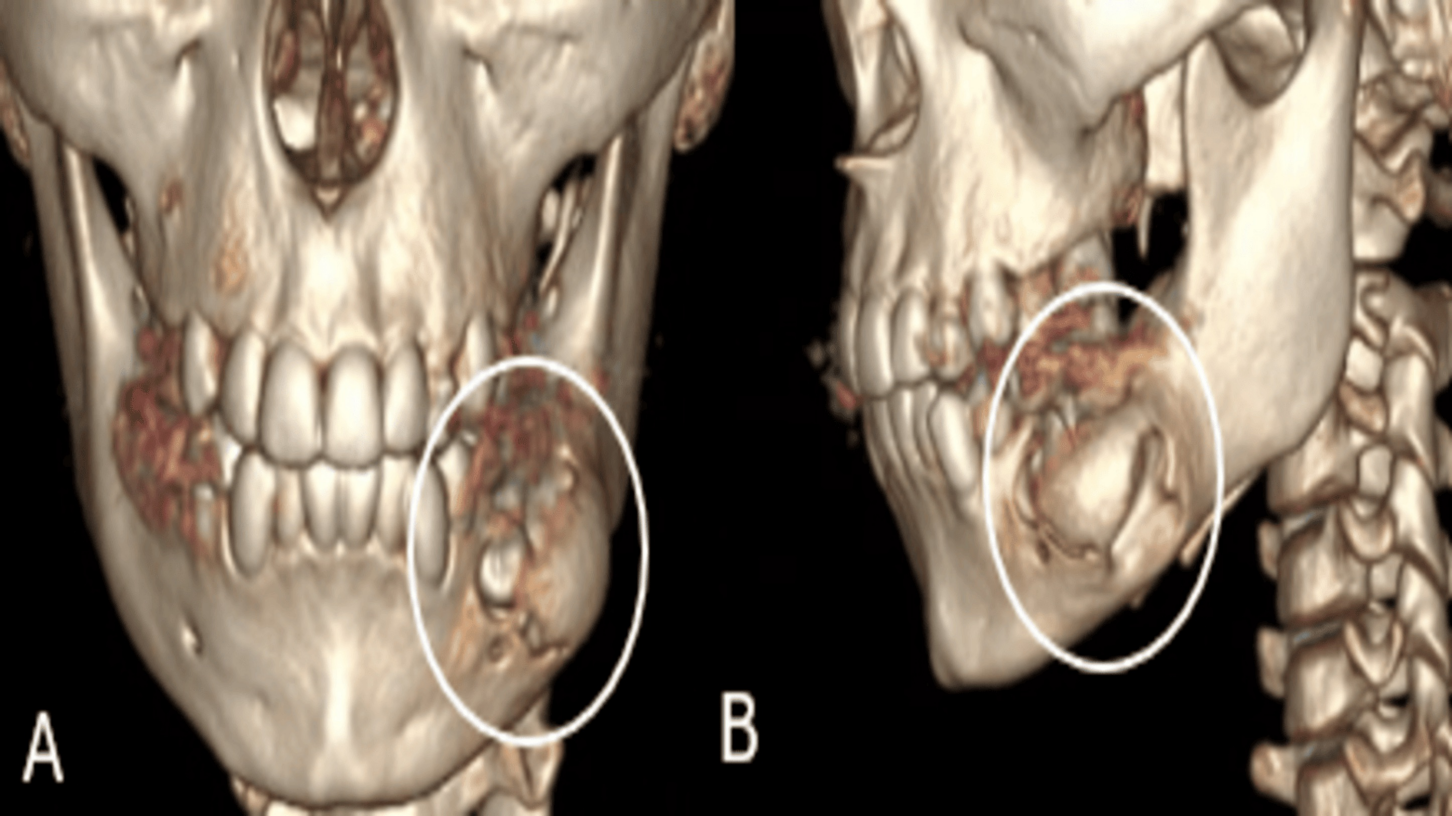
Three-dimensional CT reconstructions of the mandible in frontal (A) and lateral (B) views showing left mandibular expansion with inferior displacement of an unerupted premolar. (A) Frontal view: showing expansion of the left mandibular body caused by an underlying cystic lesion. (B) Lateral view: revealing displacement of an unerupted mandibular premolar into the inferior mandibular border.

**Figure 3:**
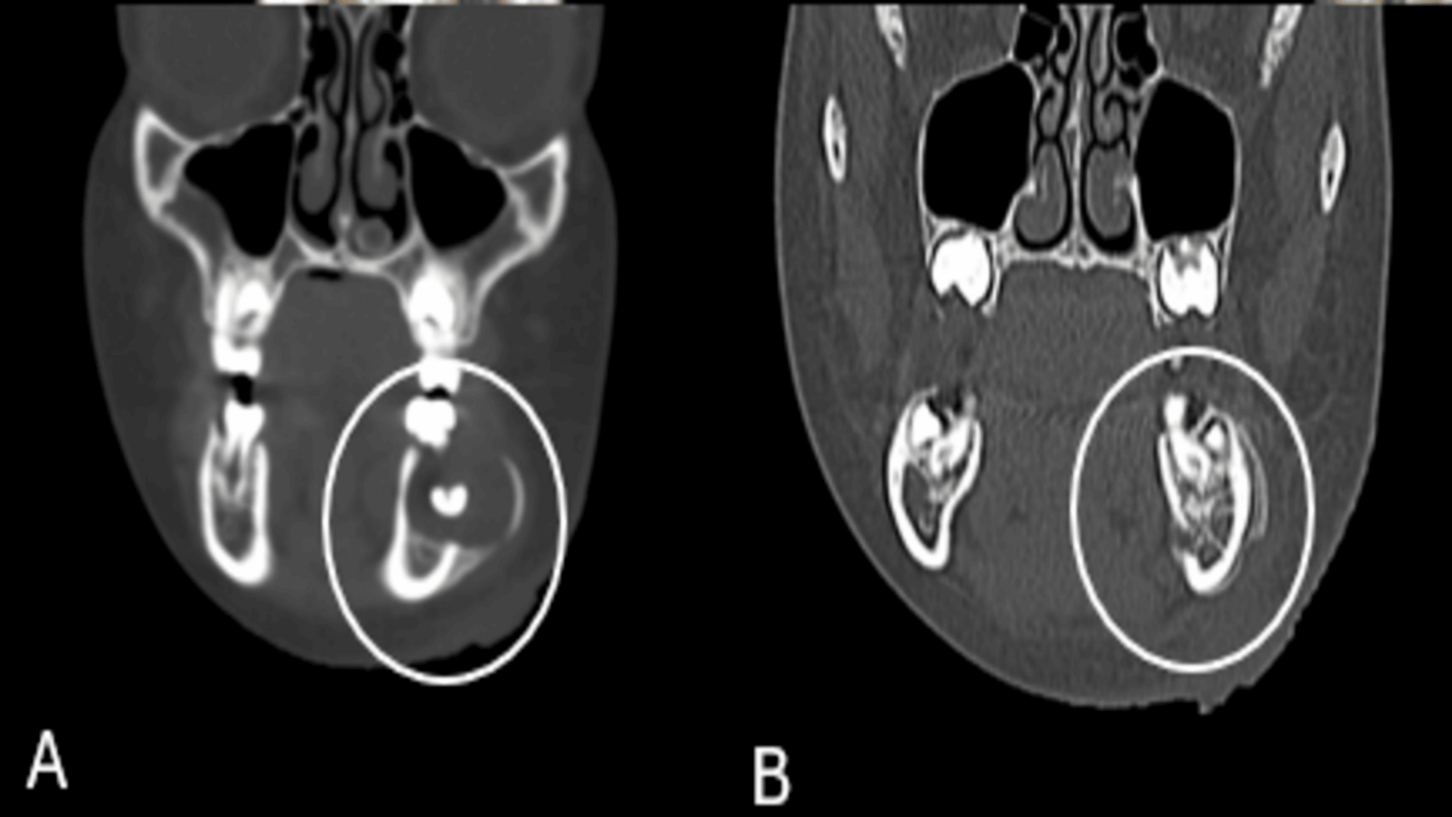
Coronal CT images of the mandible in bone (A) and soft-tissue (B) windows demonstrating a well-defined cystic lesion associated with an unerupted mandibular premolar (circled).

**Figure 4:**
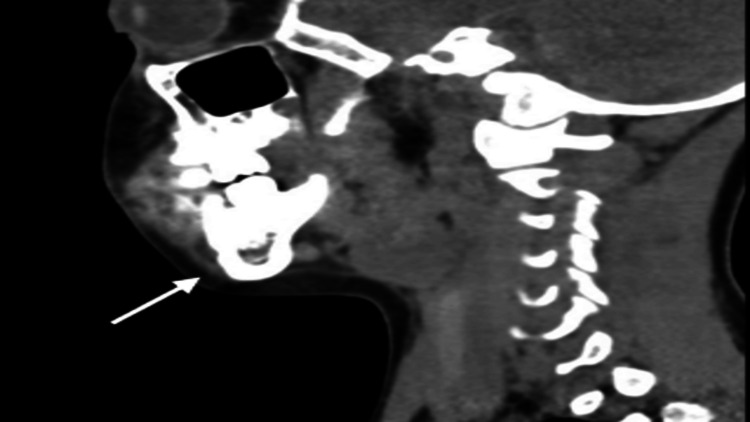
Sagittal CT (bone window) illustrating the cystic lesion’s relationship to adjacent teeth and cortical surfaces.

A neck and mandibular CT scan revealed a well-defined cystic lesion measuring 2.3 × 2.0 × 2.5 cm with periapical involvement in relation to the left mandibular first molar (Figures 2-4).

The lesion was clearly demarcated, with a thin cortical rim and partly cortically interrupted; the contents were homogeneous and hypodense. A fragment of a tooth (unerupted or underdeveloped permanent second premolar) was found at the base of the cyst. Roots of the affected molar were slightly thinned, and a localized interruption of bone was observed with a triangular periosteal reaction in the area. There were no signs of a more significant spread into the surrounding soft tissue or intraoral presentation. These findings were characteristic of periapical cysts with cystic degeneration or dentigerous cysts, particularly concerning the non-erupted tooth located in between. Opinion from the oral and maxillofacial surgery team was sought, and they recommended admitting for surgery. The patient was empirically started on intravenous clindamycin. She underwent surgical excision of the lesion under general anesthesia. A left buccal vestibular incision was performed, and a mucoperiosteal flap was elevated. The cyst was opened, and its wall was explored. The left mandibular primary second molar and impacted permanent second premolar were surgically extracted. The oral defect was closed. The left cheek extraoral fistula was excised and closed. There were no intraoperative complications and little blood loss. She remained afebrile and hemodynamically stable in the postoperative period. The culture of the wound yielded *P. oris*, an anaerobic organism typically associated with chronic odontogenic infections. Her antibiotic treatment was then switched from intravenous (IV) clindamycin to IV amoxicillin-clavulanate and from IV metronidazole, aiming for better anaerobe and Gram-negative coverage. Histopathological examination of the removed specimen demonstrated features resembling those of an inflamed odontogenic cyst of the dentigerous type, revealing a hyperplastic, non-keratinized epithelium lining, chronic inflammatory cells, granulation tissue, odontogenic rests of epithelium, and reactional osseous change. There was no dysplasia or carcinoma.

 She was discharged on oral amoxicillin-clavulanate (45 mg/kg/day divided every 12 hours) and metronidazole (10 mg/kg/dose every 8 hours) for a total duration of 7 days along with wound care. She was reviewed in the maxillofacial surgery clinic and reassured.

## Discussion

Dentigerous cysts are the second most common cysts of the jaws, accounting for 20% of all jaw cysts. Classically, they are observed around the crowns of unerupted or impacted teeth, such as mandibular third molars and maxillary canines [1, 2]. Although odontomes are most frequently encountered in late childhood and adolescence, their behavior as a clinical entity in younger children warrants special attention, as odontomes may impede tooth eruption, dislocate erupting permanent teeth, lead to pathological bone resorption, or cause long-lasting occlusal disturbances if not detected early [3, 4]. Dentigerous cysts are often asymptomatic and are discovered on dental examination. Orthodontic extraction-related X-ray studies are also always visible. However, when these lesions increase in size, they can present with a gradual onset of facial deformity, cortical bone expansion, dental mobility, and pain. Secondary infection is seldom reported but may occur, with bacteria transmitted into a carious primary tooth by oral flora or through periapical inflammation, and the cyst presenting as an uncomplicated odontogenic abscess [5, 6].

It was likely that untreated or incompletely managed caries in this patient had initiated the chain of events culminating in chronic periapical conditions. For this reason, the chronic irritation that occurred only in our case may have led to the development of a secondary infection of a pre-existing dentigerous cyst and to radiographic findings that are similar to radicular cystic lesions [5]. The presence of the impacted second premolar within the cystic cavity, as well as the pericoronal radiolucency observed on the three-dimensional CT scan, strongly suggests a dentigerous cyst origin. Radiologically described, a well-circumscribed, unilocular radiolucency with a delicate cortical lining attached to the crown of an impacted tooth is still a diagnostic hallmark that separates a dentigerous cyst from other odontogenic lesions [7]. CT, especially for children with extraoral swelling, supports imaging, as the features characterizing the lesion, its penetration through the cortex, periosteal reaction, and involvement of adjacent structures are not prominent. In the instant patient, CT demonstrated focal cortical disruption, periosteal new bone formation, and slight thinning of the adjacent molar roots, none of which are typical features of small or infected dentigerous cysts, but which can occasionally mimic a more aggressive process [7, 8].

An extraoral fistula, as seen in this child, is a rare complication of developmental odontogenic cysts but is more frequently associated with chronic periapical infections caused by necrotic primary teeth [9]. Chronic infection leads to the formation of minimal, silent tracts that extend into adjacent bone and soft tissue, resulting in a sinus on the skin. There may be a high rate of inaccurate diagnoses due to early misdiagnoses attributed to infectious and/or facial abscess etiology, and multiple empiric courses of antibiotics are ineffective [9]. The patient did have a draining fistula, which demonstrates the chronicity of the infection and the degree of local inflammation.

From a bacterial standpoint, *P. oris*, the microorganism isolated from this patient, is one of several pigmented anaerobic Gram-negative rods that are frequently isolated in association with chronic dental and endodontic infections [9]. These organisms show varying degrees of macrolide and clindamycin resistance, explaining why the child did not improve on oral antibiotics [9]. Therefore, current pediatric odontogenic infection guidelines advise broad anaerobic cover in the context of suspected or documented Gram-negative anaerobes - commonly a β-lactam/β-lactamase inhibitor combined with metronidazole [9]. The present case highlights the importance of culture-directed therapy, particularly in children failing to respond to empiric antibiotics.

The treatment of choice for dentigerous cysts depends on their size, symptoms, infection, and developmental stage of the teeth involved. In young children, marsupialization or decompression is frequently better to preserve the eruptive potential of permanent teeth [9]. However, if the cyst is infected, it has caused significant cortical disruption due to its size, and if the affected tooth becomes grossly displaced or nonvital (as in the present case), total enucleation along with the extraction of associated teeth should be considered the preferred treatment option [9]. The cyst was enucleated, and the second primary molar/impacted premolar was extracted. Both teeth were planned for extraction in view of indolent inflammatory changes, root ankylosis, and a poor long-term unerupted tooth prognosis.

Histopathology, the gold standard, confirms the diagnosis. The present case showed hyperplastic non-keratinized epithelium, chronic inflammatory infiltrate, granulation tissue, and odontogenic rest, features that are seen in an infected dentigerous cyst [2,5]. The absence of epithelial dysplasia or carcinogenesis features confirms the generally benign nature of these lesions. Infrequent malignant transformation has been reported in untreated adult patients to ameloblastoma or squamous cell carcinoma, but this condition is extremely rare in children [9].

The postoperative pediatric convalescence is usually uneventful, and recurrences are rare after complete removal of the mass [9]. Our patient had, however, a progressive decrease in swelling, an increase in oral intake, and good clinical status by 4 days postoperatively. Early maxillofacial surgery follow-up is required for wound healing checks, recurrence, and consultation regarding long-term dental rehabilitation.

In conclusion, this case highlights several advantageous principles of practice: Dentigerous cysts in children can become secondarily infected and may present as a regular abscess; ideally, the role of radiology should be emphasized to differentiate developmental-based diseases from periapical-based pathologies; anaerobic bacteria, such as *P. oris*, require organism-directed antimicrobials; finally, complete enucleation is appropriate after an acute presentation or structural compromise. In the larger picture of the problem, early dental examination and referral are crucial for such children who present with repeated jaw swelling, so that delayed diagnosis leading to potentially avoidable complications like the formation of a fistula and arrest of development of permanent teeth can be avoided.

## Conclusions

This case reports an unusual infected dentigerous cyst in a child who presented with long-standing swelling and an extraoral fistula, illustrating that recurrent or non-healing dental infection should prompt early imaging and specialist referral. Discovering an unerupted tooth within the cyst cavity, along with the diagnosis of anaerobic infection, permitted a targeted surgical and antimicrobial approach, leading to complete healing. Early identification, proper imaging, total cyst enucleation, and culture-specific antibiotics are necessary to avoid complications and hand over long-term oral health in children.
